# Oligonucleotide Arrays *vs.* Metaphase-Comparative Genomic Hybridisation and BAC Arrays for Single-Cell Analysis: First Applications to Preimplantation Genetic Diagnosis for Robertsonian Translocation Carriers

**DOI:** 10.1371/journal.pone.0113223

**Published:** 2014-11-21

**Authors:** Laia Ramos, Javier del Rey, Gemma Daina, Manel García-Aragonés, Lluís Armengol, Alba Fernandez-Encinas, Mònica Parriego, Montserrat Boada, Olga Martinez-Passarell, Maria Rosa Martorell, Oriol Casagran, Jordi Benet, Joaquima Navarro

**Affiliations:** 1 Unitat de Biologia Cel·lular i Genètica Mèdica, Facultat de Medicina, Departament de Biologia Cel·lular, Fisiologia i Immunologia, Universitat Autònoma de Barcelona, 08193, Bellaterra, Spain; 2 Qgenomics Laboratory, 08003, Barcelona, Spain; 3 Institut Universitari Dexeus, 08028, Barcelona, Spain; 4 Fundació Puigvert, Hospital de Sant Pau i de la Santa Creu, 08025, Barcelona, Spain; 5 Unitat de Reproducció Humana i Diagnòstic Genètic, Clínica Girona, 17002, Girona, Spain; 6 Genomic Service, Center for Research in Agricultural Genomics (CRAG), 08193, Bellaterra, Spain; CCR, National Cancer Institute, NIH, United States of America

## Abstract

Comprehensive chromosome analysis techniques such as metaphase-Comparative Genomic Hybridisation (CGH) and array-CGH are available for single-cell analysis. However, while metaphase-CGH and BAC array-CGH have been widely used for Preimplantation Genetic Diagnosis, oligonucleotide array-CGH has not been used in an extensive way. A comparison between oligonucleotide array-CGH and metaphase-CGH has been performed analysing 15 single fibroblasts from aneuploid cell-lines and 18 single blastomeres from human cleavage-stage embryos. Afterwards, oligonucleotide array-CGH and BAC array-CGH were also compared analysing 16 single blastomeres from human cleavage-stage embryos. All three comprehensive analysis techniques provided broadly similar cytogenetic profiles; however, non-identical profiles appeared when extensive aneuploidies were present in a cell. Both array techniques provided an optimised analysis procedure and a higher resolution than metaphase-CGH. Moreover, oligonucleotide array-CGH was able to define extra segmental imbalances in 14.7% of the blastomeres and it better determined the specific unbalanced chromosome regions due to a higher resolution of the technique (≈20 kb). Applicability of oligonucleotide array-CGH for Preimplantation Genetic Diagnosis has been demonstrated in two cases of Robertsonian translocation carriers 45,XY,der(13;14)(q10;q10). Transfer of euploid embryos was performed in both cases and pregnancy was achieved by one of the couples. This is the first time that an oligonucleotide array-CGH approach has been successfully applied to Preimplantation Genetic Diagnosis for balanced chromosome rearrangement carriers.

## Introduction

A recent systematic review including comprehensive chromosome analysis and FISH found that 22% of the *in vitro* fertilized (IVF) embryos analyzed were diploid, while 73% were mosaic: 59% were diploid-aneuploid mosaic and 14% were aneuploid mosaic, and 5% contained other numerical chromosomal abnormalities [1]. Aneuploidy is a major issue in advanced-maternal-age (AMA) patients [2] and balanced translocation carriers [3], however, it should not be underestimated in embryos from young couples with repeated implantation failures [4], recurrent miscarriages [5] or idiopathic sterility [6].

Comprehensive cytogenetic analysis techniques, such as metaphase comparative genomic hybridisation (mCGH), array-CGH (aCGH) or single-nucleotide polymorphism arrays (SNP-arrays), have allowed for the detection of whole-chromosome imbalances (aneuploidies) and segmental chromosome imbalances in cleavage-stage embryos [3],[7],[8] and blastocysts [9]. Until the application of mCGH, it had not been possible to analyse all of the chromosome complement in a single hybridisation step nor to analyse all chromosome length with a resolution of 10 Mb-20 Mb [10]. Development of Bacterial Artificial Chromosome (BAC) aCGH methodologies, with shorter procedures and more automatable analysis systems, allowed a widespread implementation of a comprehensive chromosome analysis technique to Preimplantation Genetic Screening of aneuploidies (PGS) [7],[11],[12]. Furthermore, the implementation of aCGH and SNP-arrays increased the resolution of analysis, allowing for the detection of smaller segmental imbalances [9],[13]. Partial chromosome gains and losses have been explained by non-repaired chromosome breakage and the specific breakpoints have sometimes been found to be coincident with previously described fragile sites [14],[15].

Comprehensive analysis techniques require cellular DNA amplification and, in this sense, a variety of whole-genome amplification (WGA) techniques have been validated [16]–[19]. Depending on the WGA system used (DOP-PCR or SurePlex), the comprehensive cytogenetic analysis performed (mCGH or BAC aCGH) and the type of data correction applied, variable correlations between the incidence of segmental imbalances and the blastomeres' replicative cell-stage have been detected [20],[21].

Array-CGH is currently a methodology of choice for many Preimplantation Genetic Diagnosis (PGD) applications, with the BlueGnome 24Sure BAC array being the most widely used platform in polar bodies, single blastomeres and trophectoderm cells [2],[12]. This type of array contains 3,000 BAC probes covering ≈25% of the genome, with a resolution of 5 Mb–10 Mb. Given the possibility of detecting small structural imbalances, this methodology has already been applied in PGD for translocation carriers [11].

Array-CGH based on oligonucleotide probes provides a higher resolution and coverage of the genome due to the higher amount of DNA probes, which are smaller in length. Recently, an oligonucleotide aCGH containing from 60K to 1 M probes per sample has been successfully validated for single-cell analysis [22],[23] and applied to PGS on single blastomeres [24] and trophectoderm cells [25].

In the present work, an oligonucleotide aCGH approach has been compared to mCGH and BAC aCGH methodologies in terms of equivalence of the cytogenetic profiles obtained in single fibroblasts and isolated blastomeres, time consumption and costs. Moreover, the use of this oligonucleotide aCGH approach in PGD cycles for Robertsonian translocation carriers was also a challenge of this work.

## Materials and Methods

### Single fibroblasts analysis

The amplified DNA (see Cell lysis and DNA amplification procedures below) from 15 single fibroblasts from different cell-lines (Coriell, New Jersey) was analyzed by mCGH and oligonucleotide aCGH: eight 47,XY,+15 isolated fibroblasts from cell-line GM03184, four 48,XY,+2,+21 isolated fibroblasts from cell-line GM03676 and three 47,XY,+13 isolated fibroblasts from cell-line GM03330. Only fibroblasts displaying the expected aneuploidy in the mCGH profiles were included in the present study.

### Human blastomeres analysis from donated cryopreserved embryos and from previously performed PGD cases

Authorisation from the *Comisión Nacional de Reproducción Humana Asistida* (CNRHA) in Spain was specifically obtained to process cryopreserved embryos from families that discarded using them for reproductive purposes and all of the families involved signed an authorised informed consent. PGD cycles were approved by each center's Ethics Committee and the families involved signed the corresponding informed consent.

Aliquots of amplified DNA from single blastomeres (see Cell lysis and DNA amplification procedure below) were sequentially analyzed by two different techniques: mCGH and oligonucleotide aCGH; or BAC aCGH and oligonucleotide aCGH.

a) Firstly, an aliquot from the amplified DNA of nine isolated blastomeres from human cryopreserved embryos, previously analyzed by mCGH (included in Ramos et al., 2013), were analyzed by oligonucleotide aCGH (Table 1).

**Table 1:** Cytogenetic results obtained from human single blastomeres analyzed by mCGH and oligonucleotide aCGH.

	mCGH			Oligonucleotide aCGH			
BL	SC	A	S	SC	A	S	Concordance
1	46,XY	-	-	46,XY	-	-	Complete
2	46,XY	-	-	46,XY	-	-	Complete
3	45,XX	−4	+13qcenq13, −13q13qter	45,XX	−4	+13qcenq13.2, −13q13.2qter	Complete
4	45,XX	−4	−8q22.1qter, +13qcenq13, −13q13qter	45,XX	−4	−8q21.2qter, +13qcenq13.2, −13q13.2qter	Complete
5	46,XY	-	-	46,XY	-	-	Complete
6	46,XY	-	-	46,XY	-	-	Complete
7	46,XY	-	-	46,XY	-	-	Complete
8	46,XY	-	-	46,XY	-	-	Complete
9	46,XY	-	−2q35qter, −4p, −11p14pter, −12p	44,XY	−11, −12	−2q35qter, −4p	A/S divergence
10	40,XXX	+1, −2, +3, −4, +5, +6, +7, −8, +9, −10, +11, −12, −13, −14, +15, −16, −17, −18, −20, −21, −22, +X	-	33,XX	−2, −4, −8, −10, −12, −13, −14, −16, −17, −18, −20, −21, −22	-	Partial
11	46,XY	−2, +3, −5, +6, −8, −11, +12, +14, +15, +16, +17, −18, −21, −22	-	39,XY	−2, −5, −8, −11, −18, −21, −22	+6q	Partial, A/S divergence
12	48,XX	+10, +13	−2q21.3qter	48,XX	+10+13	−2q21.3qter, −5p15.2pter, −18q12.2qter	Extra segments
13	50,XY	−2, +3, +5, +7, +8, +13, +16, −20	−1p21pter, +1q21.3qter, +12pterq21.3, −12q23qter, +19q	41,XY	−2, +5, +7, −14, −15, −17, −20, −21, −22	−1p21.1pter, −4q, −9q, −10q, −12q23.1qter, −18q	Partial
14	47,XX	+14	+9q	47,XX	+14	−6p12.3p22.2, +9qcenq33.1	Extra segments
15	45,XX	−18	-	45,XX	−18	−5p15.1pter	Extra segments
16	46,XX	-	−2q24.3qter	46,XX	-	−2q24.1qter, −15q21qter	Extra segments
17	46,XY	+13, −14	+20p	42,XY	−14, −15, −16, −17	−8p, +13q21qter	Partial, A/S divergence
18	45,XY	−15	-	45,XY	−15	-	Complete

BL: Blastomere; SC: Sexual chromosomes, A: Aneuploidies; S: Segmental imbalances.

Cells 10-13: Globozoospermia PGD case, cells 14-18: Robertsonian translocation carrier PGD case (45,XY,der(13;14)(q10;q10)).

b) Afterwards, aliquots from the amplified DNA of nine isolated blastomeres from previous PGDs performed in collaboration with *Fundació Puigvert*, were analyzed by mCGH and oligonucleotide aCGH (Table 1).

c) Finally, an aliquot from the amplified DNA of sixteen isolated blastomeres from previous PGDs performed at the *Institut Universitari Dexeus* by BAC aCGH, were analyzed by oligonucleotide aCGH (Table 2).

**Table 2:** Cytogenetic results obtained from human single blastomeres analyzed by BAC aCGH and oligonucleotide aCGH.

	BAC aCGH			Oligonucleotide aCGH			
BL	SC	A	S	SC	A	S	Concordance
19	48,XY	+16, +17	-	48,XY	+16, +17	-	Complete
20	51,X	+1, −2, +6, +7, +8, −9, +11, +12, +14, +15, +16, +18, −20, −X/Y	-	49,X	+1, −2, +6, +8, −9, +11, +14, +16, +19, −20, −X/Y	+7pcenp15, +12q, −13q14.12q33.3, +15qcenq25.2, −15q25.3qter, +18q	Partial, A/S divergence
21	45,XX	−16, +21, −22	-	45,XX	−16, +21, −22	-	Complete
22	47,XX	+15	-	47,XX	+15	-	Complete
23	46,XY	-	−6q	46,XY	-	−6q	Complete
24	46,XX	-	-	46,XX	-	-	Complete
25	39,XY	−1, +3, −4, −6, −7, −8, −9, −13, −22	-	39,XY	−1, +3, −4, −6, −7, −8, −9, −13, −22	-	Complete
26	49,XX	+12, +13, +16	-	48,XX	+12, +13	+16p	A/S divergence
27	45,XX	−10	-	45,XX	−10	-	Complete
28	49,XY	+1, +2, +3, −8, +10	-	49,XY	+1, +2, +3, −8, +10	−9q34.1qter	Extra segments
29	47,XY	+22	-	47,XY	+22	-	Complete
30	46,XX	-	-	46,XX	-	-	Complete
31	46,XX	-	-	46,XX	-	-	Complete
32	49,XY	+1, −2, −4, +11, +13, +17, +18	-	49,XY	+1, −2, −4, +11, +13, +17, +18	-	Complete
33	46,XY	-	-	46,XY	-	-	Complete
34	46,XX	-	-	46,XX	-	-	Complete

BL: Blastomere; SC: Sexual chromosomes, A: Aneuploidies; S: Segmental imbalances.

Cells 19-34: PGDs for six different couples with either male factor or repeated implantation failures.

### PGDs for Robertsonian translocation carriers

Two PGDs for Robertsonian translocation carriers 45,XY,der(13;14)(q10;q10) were performed in collaboration with *Fundació Puigvert*, in Barcelona (Spain). Both PGD cycles were approved by the center's Ethics Committee and the families involved signed the corresponding informed consent.

The first patient (A) had a severe teratozoospermia, with a Sperm DNA Fragmentation (SDF) of 29% measured with the Sperm Chromatin Dispersion test (SCD test), single-strand SDF (ssSDF) of 62.8% and double-strand SDF (dsSDF) of 94% (ssSDF and dsSDF were measured with the alkaline and neutral Comet assay, respectively). The second patient (B) had an oligozoospermia, with a SDF of 39%, ssSDF of 59% and dsSDF of 54%. The SCD test was performed using the Halosperm kit (Halotech DNA; Madrid, Spain) following the manufacturer's instructions. The alkaline and neutral Comet assays were performed as previously described [26].

Females (46,XX) were 29 y.o and 31 y.o, respectively. They underwent routine superovulation procedures and embryos were fertilised on Day 0 by intracytoplasmic sperm injection (ICSI). On day 3 after fertilisation, one blastomere from each evolved embryo that had reached the 6-8-cell stage was biopsied using Tyrode's acid. The comprehensive cytogenetic analysis was performed with oligonucleotide aCGH and a re-analysis was performed by mCGH. The available discarded embryos were analyzed on Day 5 by mCGH.

### Cell lysis and DNA amplification

All of the single cells were lysed and their DNA was amplified following the SurePlex DNA Amplification System (BlueGnome, Cambridge, UK), according to the manufacturer's instructions. Electrophoresis on a 1.5% agarose gel was used to evaluate the correct DNA amplification of each sample (smears between 200pb and 1500pb).

### Oligonucleotide aCGH

The SurePrint G3 Human CGH Microarray Kit (Agilent Technologies, Santa Clara, California) was used following the manufacturer's instructions. Set-up of the methodology was performed with the Agilent protocol for genomic DNA (array formats 1x1M, 2x400K, 4x180K and 8x60K) and the protocol for single-cell analysis (array format 8x60K).

Both PGD applications (A and B) were performed with Agilent single-cell methodology using 13 µl of SurePlex amplification in 8x60K arrays with 16-hour hybridisation, following the manufacturer's instructions. In the PGD performed to couple A, hybridisation of the same DNA amplification product was also performed on 4x180K arrays to set up the Agilent single-cell protocol in this array format. A volume of 26 µl from each DNA amplification product was labelled by adding 5 µl of *Random primer* to each tube and incubating this 3 min at 95°C and 5 min at 4°C; 10 µl of 5x *Reaction buffer*, 5 µl of *10x dNTPs*, 3 µl of *Cy5-dUTP* and 1 µl of *Klenow* enzyme were added, and incubation for 2 h at 37°C and 10 min at 65°C was performed. The same procedure was followed for the reference DNA. After purification of the labelled samples, test and reference DNA were mixed (1∶1) with 5 µl *Cot-DNA* (Roche), 11 µl of *Blocking agent* and 55 µl of *2x HI-RPM Hybridisation Buffer*. A total of 100 µl from each sample were hybridised. Sixteen-hour hybridisation was performed at 65°C and 20 rpm in the Agilent Hybridisation Oven.

Workbench Standard Edition software (Agilent) was used for the cytogenetic analysis. The ADM-2 algorithm was applied for analysis and a threshold of 10 was used for determining cytogenetic abnormalities. A minimum of 10 consecutive gained or lost probes was required to describe an unbalanced region.

### BAC aCGH

SurePlex 24sure V3 CGH arrays (BlueGnome) were used following the manufacturer's instructions. BlueFuse Multi software (BlueGnome) was used for the cytogenetic analysis.

### mCGH

The CGH procedure was performed as previously described [27]. Analysis was performed capturing 12 metaphases per sample with a Nikon 90i epifluorescence microscope (Nikon, Tokyo, Japan). Evaluation of the hybridisation was made using the Isis CGH software (MetaSystems, Altlussheim, Germany). To diagnose chromosome gains and losses, thresholds were fixed at 0.8 and 1.2, respectively.

### Interpretation criteria

For the three approaches described, chromosome 17, 19 and 22 aneuploidies have been considered as being technical artifacts when all three chromosomes were simultaneously gained or lost in the same cell, as previously described [28],[29]. Deviation of 1p33pter was also considered to be artifactual, as previously described [15].

## Results

### Set-up of the Agilent oligonucleotide aCGH

Agilent protocol for genomic DNA analysis was validated for single-cell analysis using amplified DNA product from single fibroblasts with all available slide formats (1x1M, 2x400K, 4x180K, 8x60K). The Agilent single-cell protocol was also validated with 8x60K arrays. The known abnormalities from the three cell lines used were detected by both mCGH and oligonucleotide aCGH in 100% of the fibroblasts analyzed (15 out of 15) (Figure S1).

Re-analysis of 34 blastomeres previously analyzed by mCGH or BAC aCGH gave broadly similar cytogenetic diagnoses using oligonucleotide aCGH in all of the cases (Tables 1 and 2).

**Figure 1:**
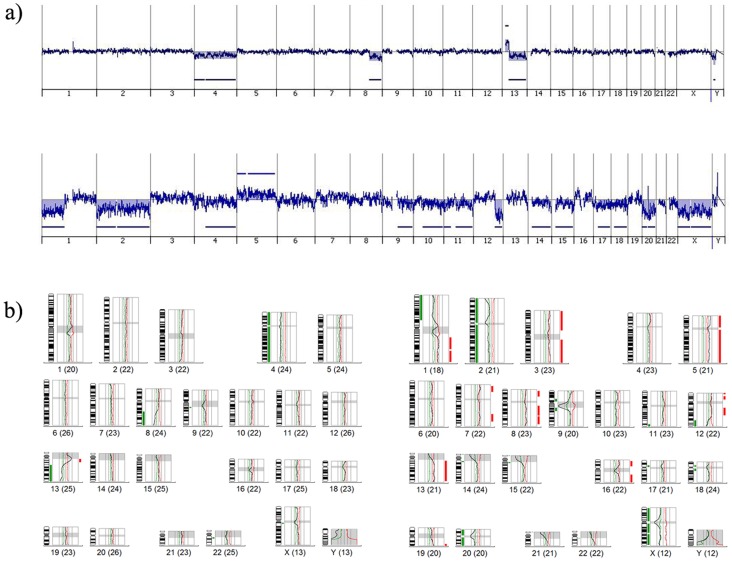
Examples of a) oligonucleotide aCGH profiles and b) their corresponding mCGH profiles. The first profile from a) and b) correspond to one blastomere with totally coincident profiles between techniques, and the second profile from a) and b) correspond to one blastomere with highly similar cytogenetic results.

Of the 18 blastomeres previously analyzed by mCGH, nine showed completely coincident cytogenetic results when analyzed by oligonucleotide aCGH (Fig. 1); four showed extra segmental imbalances; four were partially coincident as, although showing extensive aneuploidies, different chromosomes were involved depending on the method used (Fig. 1); and three showed aneuploidies for chromosomes that, using mCGH, showed segmental imbalances, or *vice versa* (Table 1).

**Figure 2:**
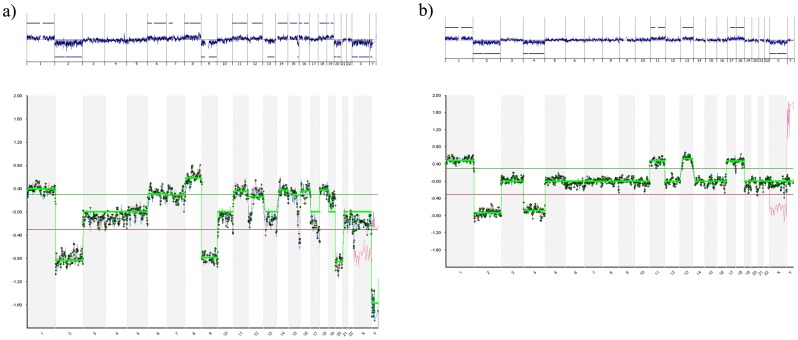
Examples of a) totally coincident profiles and b) highly similar profiles, obtained with oligonucleotide aCGH and BAC aCGH. Oligonucleotide aCGH profiles are shown at the top of the figure and BAC-aCGH profiles are shown below.

Of the 16 blastomeres previously analyzed by BAC aCGH, 13 showed totally coincident cytogenetic results when analyzed by oligonucleotide aCGH (Fig. 2a); one showed extra segmental imbalances; one was partially coincident as, although showing extensive aneuploidies, different chromosomes were involved depending on the method used; and two showed segmental imbalances with oligonucleotide aCGH that were detected as aneuploidies with BAC aCGH (Fig. 2b) (Table 2).

### PGD for Robertsonian translocation carriers

To our knowledge, this is the first time that PGD has been successfully performed to balanced Robertsonian translocation carriers following an oligonucleotide aCGH approach.

Two PGD cases were performed analysing single blastomeres from a total of 17 evolved embryos (9 from couple A and 8 from couple B) with the Agilent single-cell protocol in 8x60K arrays. Both, the segregation pattern of the rearranged chromosomes and the presence of aneuploidies and segmental chromosome imbalances involving the rest of the chromosomes, were identified in each diagnosed embryo. The type of meiotic segregation from the trivalent figure was inferred in 16 out of 17 embryos: 10 out of 16 were normal or balanced for the chromosomes involved in the rearrangement (2∶1 alternate segregation) and 6 out of 16 resulted from an abnormal segregation (2∶1 adjacent segregation). Aneuploidies not related to the rearranged chromosomes and/or segmental imbalances were detected in 11 out of 16 embryos (68.7%). Transfer of the three cytogenetically normal embryos was performed and pregnancy was achieved by couple B (Table S1).

Re-analysis of the same amplified DNAs by mCGH were totally coincident with the results previously obtained with 8x60K arrays in 14 out of 16 embryos (Table S1). Re-analysis of the same amplified DNAs by the Agilent single-cell protocol applied to 4x180K arrays were totally coincident in 7 out of 9 embryos from couple A (Table S1).

Analysis of the discarded embryos confirmed the type of meiotic segregation from the trivalent figure in 10 out of 12 embryos (Table S2). Divergent profiles from the single blastomere from E6 (couple A), analyzed in the PGD application, and the whole discarded embryo are shown in Figure S2.

## Discussion

The use of oligonucleotide array-CGH has not been widely implemented in PGD routine as it had only been applied before in a couple of centers for PGS [24],[25]. Nevertheless, most PGD and PGS applications are nowadays performed following BAC array CGH platforms [2],[11],[12].

In the present work, three of the available methodologies for single-cell comprehensive cytogenetic analysis have been compared. The less commonly used approach, the oligonucleotide aCGH, has been set up providing broadly similar cytogenetic results to those obtained with mCGH and BAC aCGH. In most of the cells analyzed, the methodologies tested provided equivalent, if not identical, cytogenetic profiles. However, the oligonucleotide aCGH approach was able to better define the specific unbalanced chromosome segments due to an increased number of probes covering most of the chromosome regions, and it informed of additional segmental imbalances in five of the blastomeres analyzed (Tables 1 and 2). The smallest segmental imbalance detected with oligonucleotide aCGH comprised 11 Mbp (Blastomere 28, Table 2), length within the described theoretical resolution (≈20 kb). Only the aCGH methodologies are able to determine the specific chromosome breakpoints generating each segmental imbalance, being the oligonucleotide aCGH the technique with a higher resolution.

Different imbalanced profiles were found in five blastomeres showing extensive aneuploidies depending on the technique used (Tables 1 and 2). The presence of extensive aneuploidies in these cells and the fact that each analysis' software has its own thresholds to determine gains and losses may explain the differences observed. When only a limited number of aneuploid chromosomes were present (i.e. in the fibroblasts from cell lines), both analysis systems provided the same cytogenetic results.

The fact that equivalent cytogenetic results between oligonucleotide aCGH and the previously established techniques for the comprehensive chromosome analysis were obtained supported its application in PGD cycles. To our knowledge, this is the first time that PGD for balanced Robertsonian translocation carriers has been successfully performed following an oligonucleotide aCGH approach.

Both the chromosome segregation pattern and the presence of other aneuploidies and segmental chromosome imbalances have been assessed in each analyzed blastomere. The meiotic segregation pattern was identified in all of the analyzed embryos, showing that the 2∶1 alternate segregation was the most frequently produced (Table S1), in agreement with previously reported works [3],[30].

A total of 68.7% of the embryos analyzed showed aneuploidies and segmental imbalances not related to the rearranged chromosomes (Table S1). Since in these PGD applications the maternal age was not a risk factor potentially responsible for the increase of meiotic chromosome imbalances, the high rate of aneuploidy found could be attributable to an inter-chromosomal effect (ICE), which has been widely described in previous PGD results from Robertsonian translocation carriers [3],[31],[32]. However, the aneuploidies observed could have also been produced as a consequence of mitotic segregation errors produced during the first embryo divisions. Eighteen out of the 23 limits of the segmental imbalances observed involved common fragile sites (78.3%), so it could be inferred that most of the chromosome breaks generated during the early embryo divisions may occur in these fragile sites as a consequence of the replication process, which leads to a chromosome instability period [14],[15],[33].

Re-analysis of the discarded embryos, however, revealed that E6 from couple A was, in fact, originated by a 2∶1 adjacent segregation gamete misdiagnosed in PGD. The presence of other highly amplified chromosome segments in this particular biopsied blastomere for PGD made it hard to detect a single-copy chromosome gain (+14) by both mCGH and aCGH analysis software (Figure S2). E4 from Couple B also showed a non-coincident meiotic pattern in its re-analysis due to being a chaotic embryo: an extensive aneuploidy was observed in the PGD while two segmental imbalances were observed in another blastomere from the discarded embryo.

Only four out of the 12 re-analyzed embryos on Day 5 showed cytogenetic abnormalities not related to the chromosome rearrangement (33.3%), while six had non-balanced chromosome complements and three were totally euploid (Table S2). This can be explained by the fact that only principal abnormalities present in the blastocyst can be detected, since complementary abnormalities caused by mitotic non-disjunction would be compensated among cells. This is in agreement with an increased euploidy rate previously detected in embryos reanalysis at the blastocyst stage [34]–[36].

Both males showed sperm DNA fragmentation patterns consistent with a bad prognosis for the IVF outcome due to their high single-strand sperm DNA fragmentation, as previously described [26],[37]. The fact that Couple B achieved pregnancy after this PGD cycle lets us postulate that whole chromosome analysis in PGD is a powerful tool to be considered by couples showing this type of male infertility profile.

Additionally, it would be of interest for PGD centers to have the information related to each comprehensive chromosome analysis technique available; mainly, their resolution, time consumption and cost in order to properly inform the candidate families (Table 3). The results obtained in this work show that no strong differences in the cytogenetic results are present between mCGH and aCGH methodologies, as all of them have shown a high degree of accuracy in detecting not only aneuploidies, but also segmental imbalances. The higher resolution given by oligonucleotide aCGH, however, may provide a great advantage when compared to BAC aCGH or mCGH in applications such as prenatal diagnosis of pathologic copy-number variants [38].

**Table 3:** Resolution, procedure duration and costs of mCGH, BAC aCGH and oligonucleotide aCGH (8x60K and 4x180K formats).

Parameter	mCGH	Agilent aCGH 8x60K	Agilent aCGH 4x180K	24 sure V3 BAC aCGH
Resolution (Mb)	10–20	≈0.02	≈0.02	5–10
Cost per sample (€)	64	480	815	132
Protocol (h)	9	8.5	8.5	7.5
Hybridisation step (h)	12	16	16	12
Analysis/sample (min)^1^	60	10	10	10

One person performing analysis.

Metaphase-CGH and both aCGH procedures described can be performed by instructed technicians and have equivalent duration. Time of analysis, however, is greatly shortened with the automatized scanning of array platforms and the analysis software used (Table 3). In the three methodologies described, and consequently in both PGD cases performed, cytogenetic results are obtained on Day 4, enabling embryo transfer in the same IVF cycle [11],[12],[24],[27].

Regarding the cost of each methodology, array methodologies have higher material costs, however, optimisation of the cytogenetic analysis procedure and the higher resolution obtained may influence couples and PGD centers to opt for aCGH methodologies (Table 3).

In conclusion, an oligonucleotide aCGH methodology has been validated for single-cell cytogenetic analysis and it has been successfully applied for the first time in PGD for Robertsonian translocation carriers. Moreover, this comprehensive chromosome analysis system allowed for embryo transfer in both of the PGD cases performed and pregnancy was successfully achieved by one of the couples. The three methodologies tested provide highly comparable cytogenetic results in single cells analysis, however, both aCGH approaches show convenient advantages when compared to the mCGH: they provide an increased resolution and an optimized analysis procedure with lower time consumption. Particular advantages and limitations of each method have been evaluated in order to better choose the most suitable approach for PGD and PGS indications.

## Supporting Information

Figure S1 **Oligonucleotide aCGH profiles obtained for the 15 fibroblasts analyzed.**
(PDF)

Figure S2 **mCGH profiles obtained for E6 (Couple A) in a) one blastomere, and b) the whole, discarded embryo.**
(TIF)

Table S1 **Cytogenetic results obtained in the PGD for couples A and B, with both males carrying a 45,XY,der(13;14)(q10;q10) Robertsonian translocation.**
(DOC)

Table S2 **Cytogenetic results obtained by mCGH from the discarded embryos of the PGD for couples A and B, with both males carrying a 45,XY,der(13;14)(q10;q10) Robertsonian translocation.**
(DOC)
