# Supplementary figures and images for: Oligonucleotide Arrays vs. Metaphase-Comparative Genomic Hybridisation and BAC Arrays for Single-Cell Analysis: First Applications to Preimplantation Genetic Diagnosis for Robertsonian Translocation Carriers
Source: PLoS One. 2014 Nov 21;9(11):e113223. doi: 10.1371/journal.pone.0113223 (PMC4240610; doi:10.1371/journal.pone.0113223)

Cell A.1

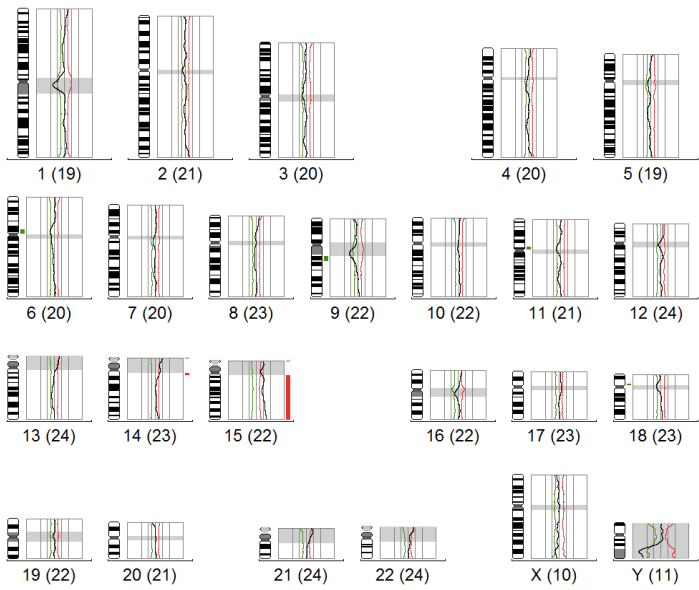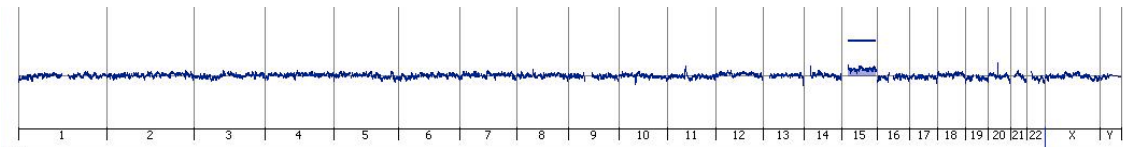

Cell A.2

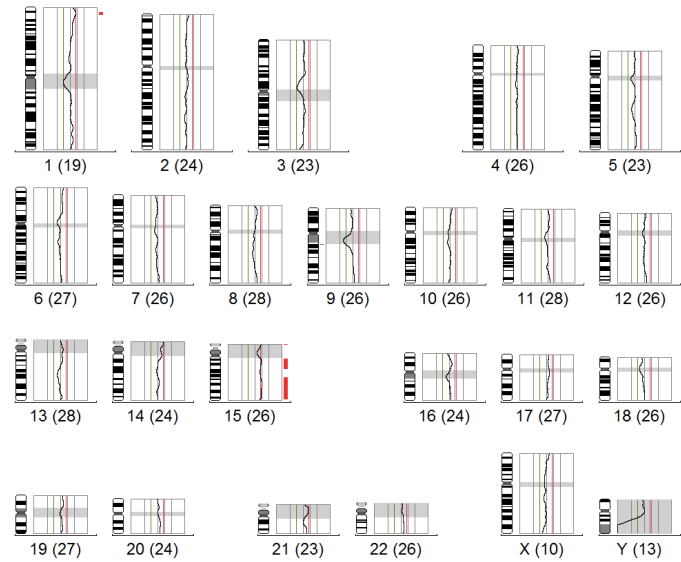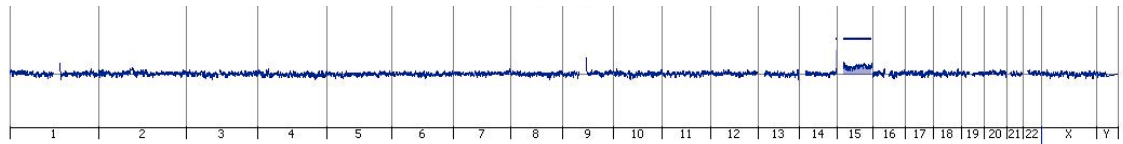

Cell A.3

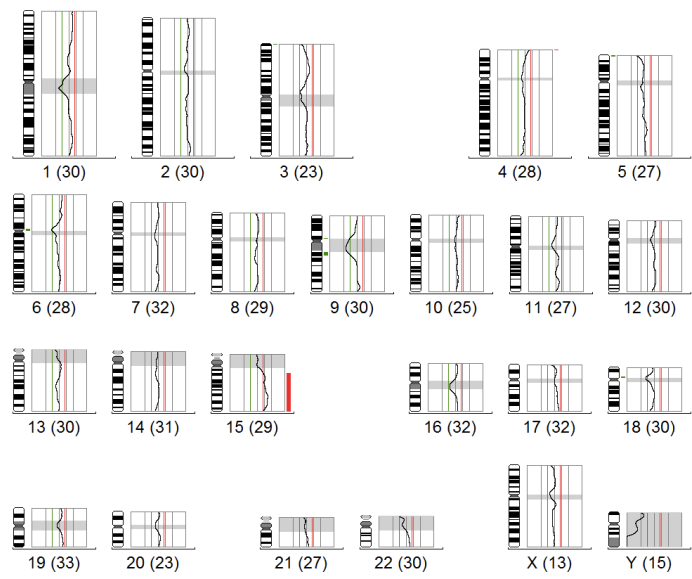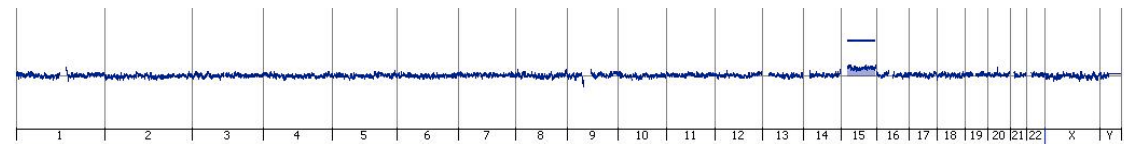

Cell A.4

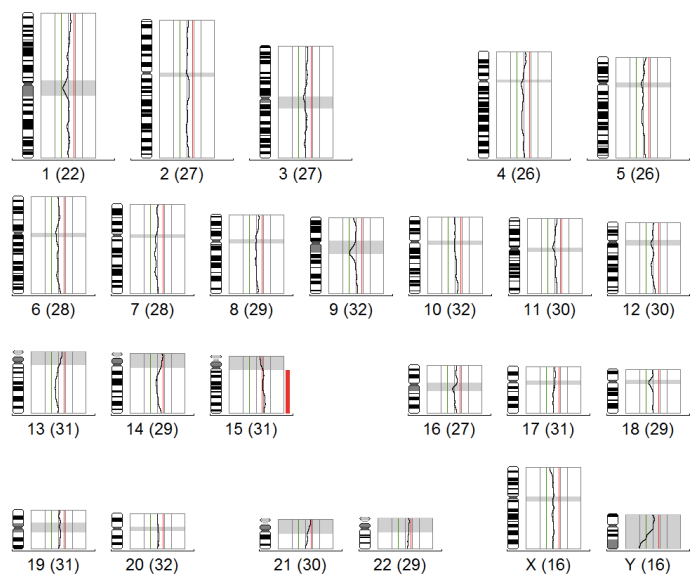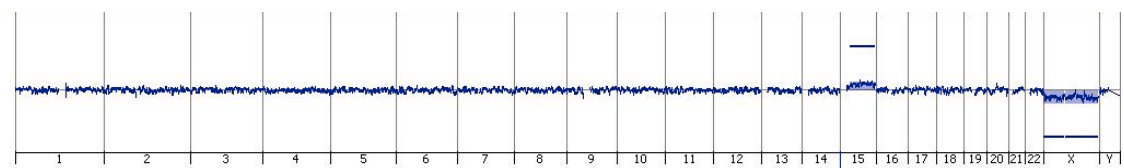

Cell A.5

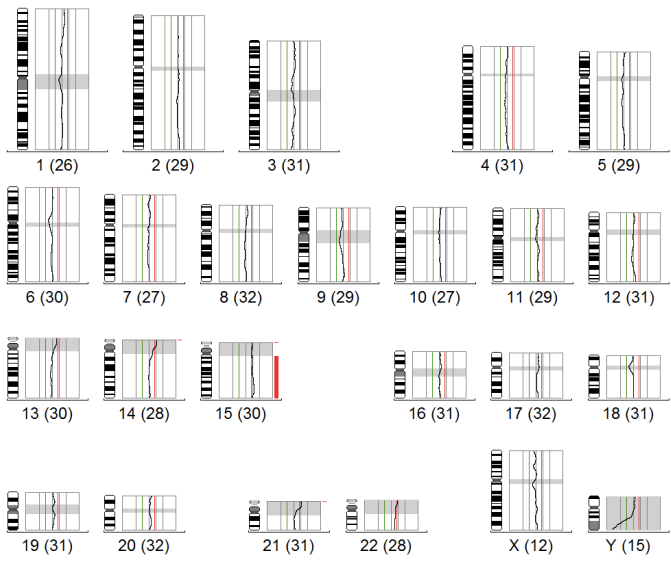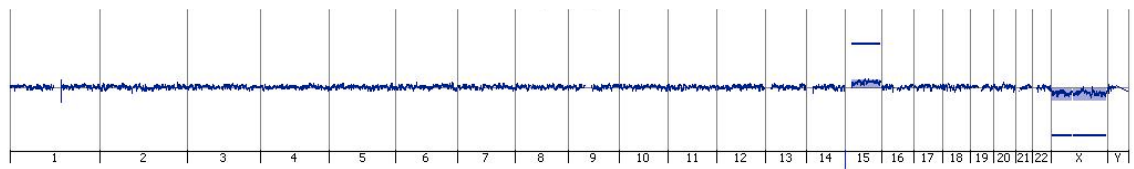

Cell A.6

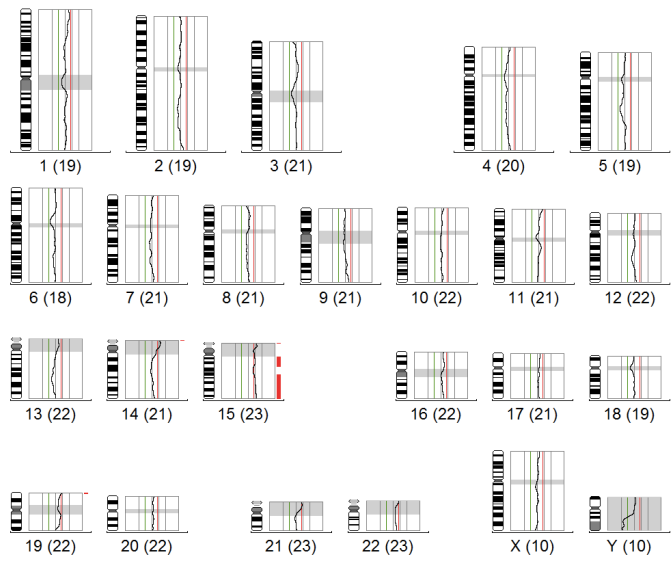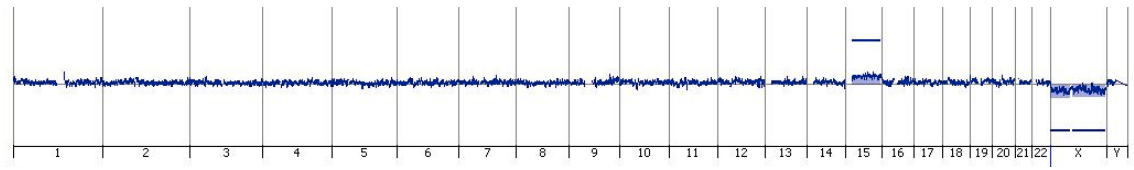

Cell A.7

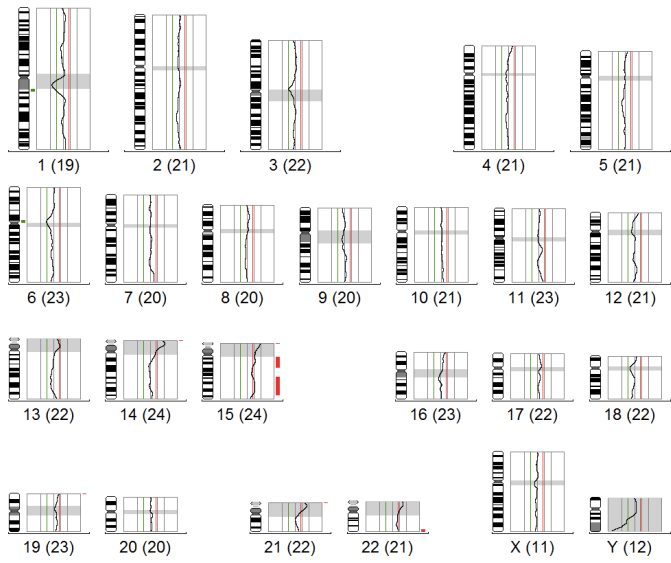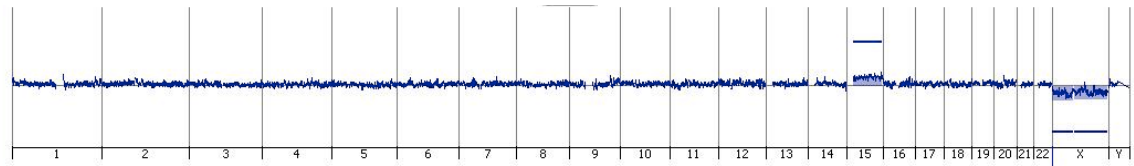

Cell A.8

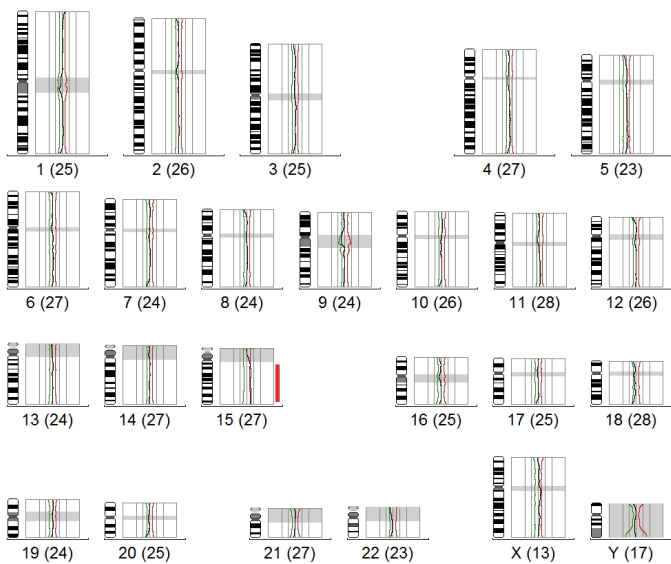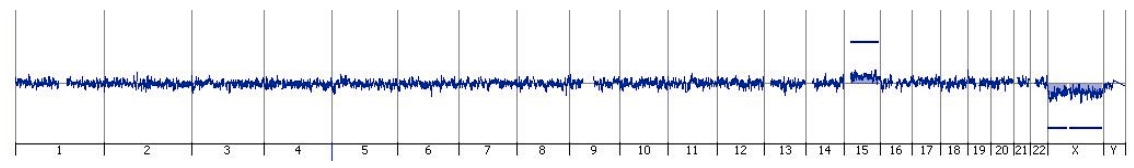

Cell B.1

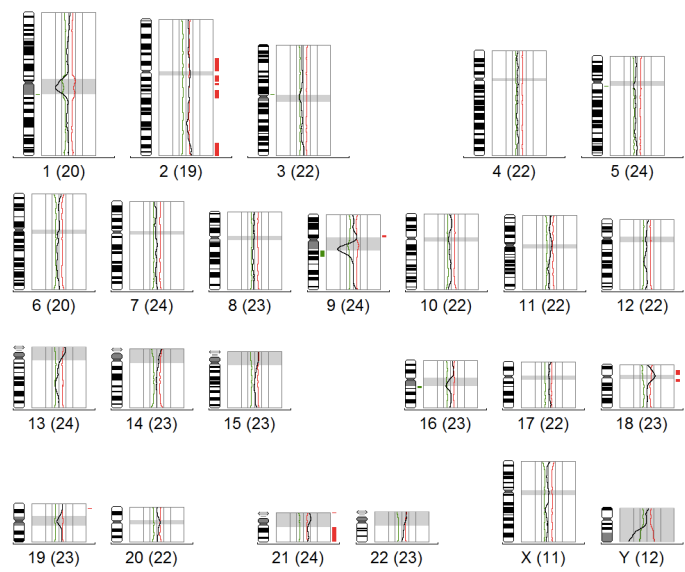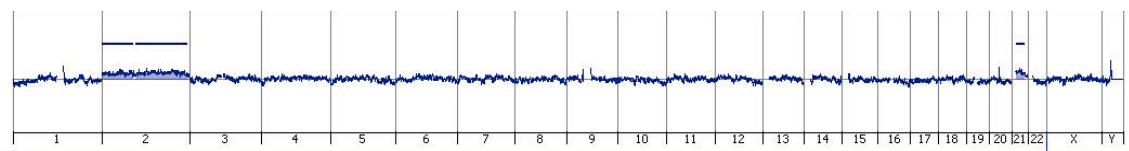

Cell B.2

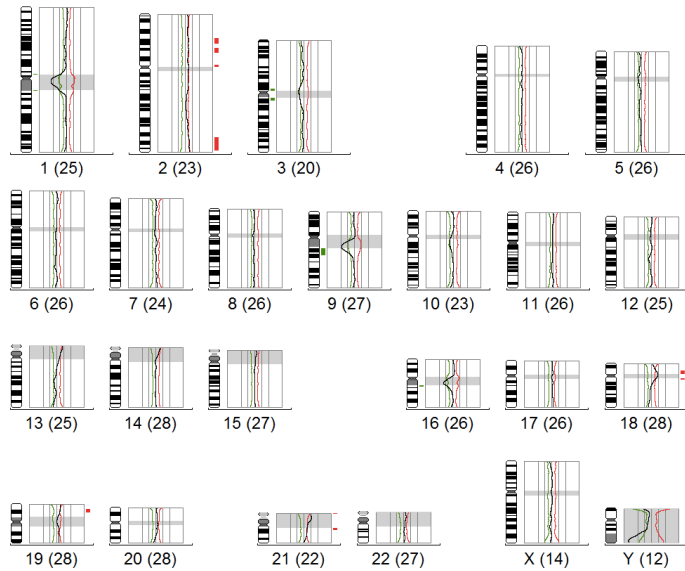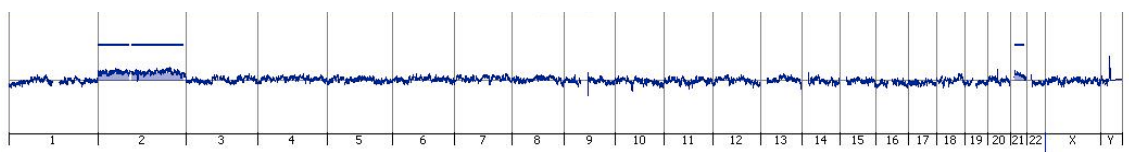

Cell B.3

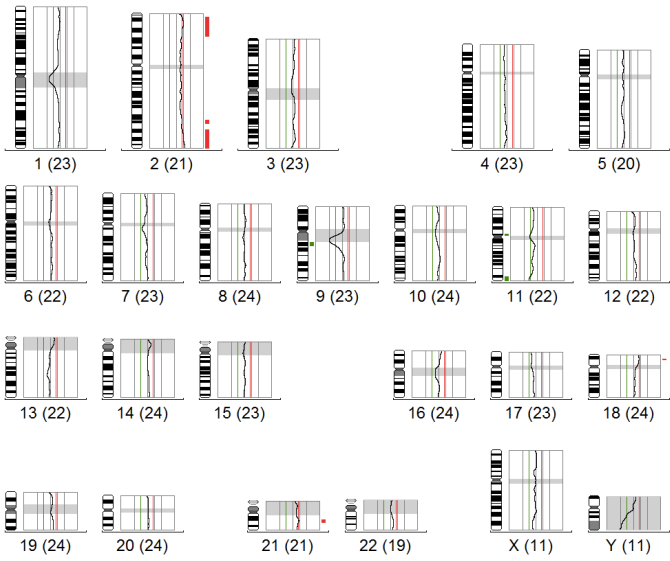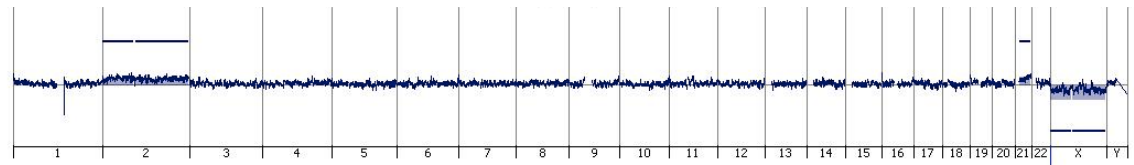

Cell B.4

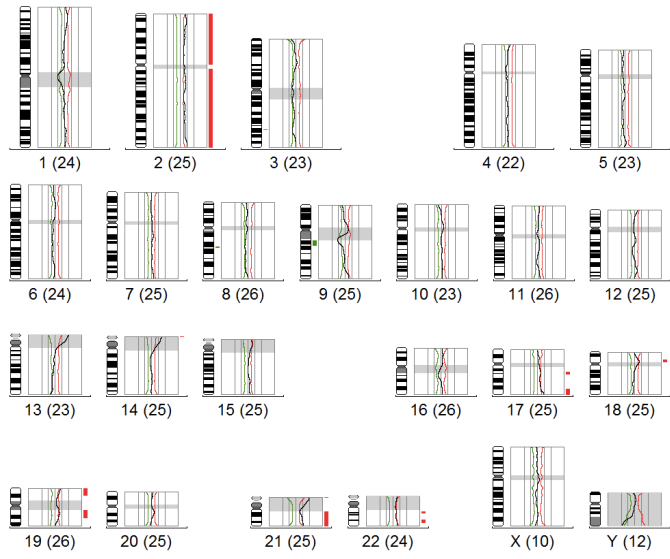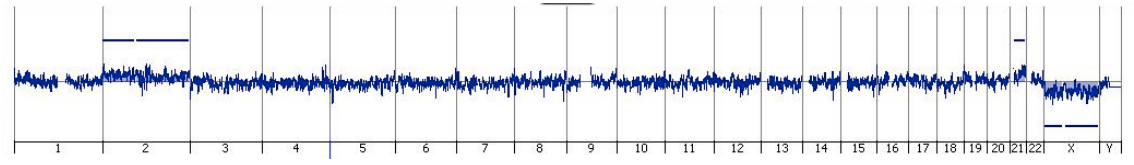

Cell C.1

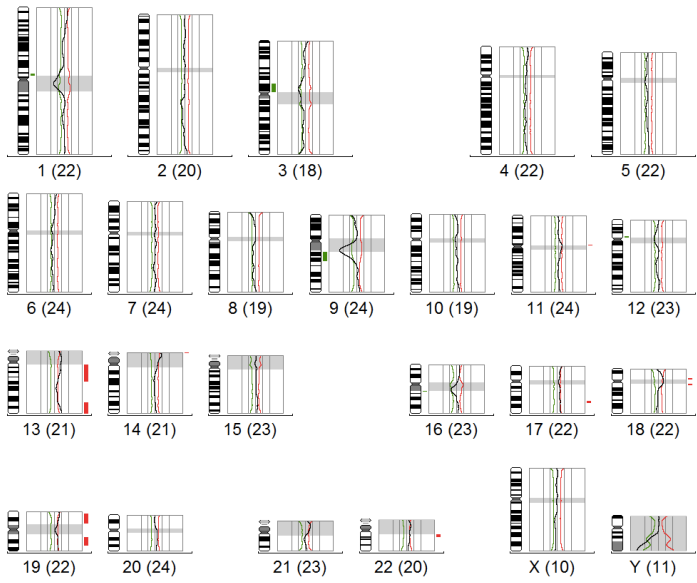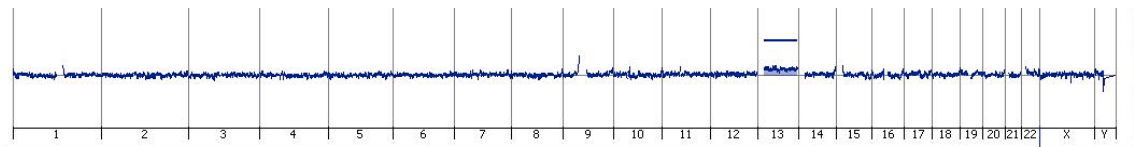

Cell C.2

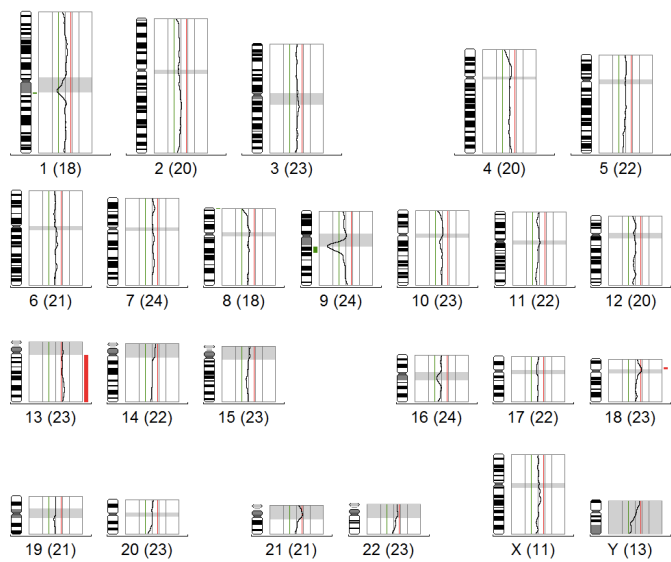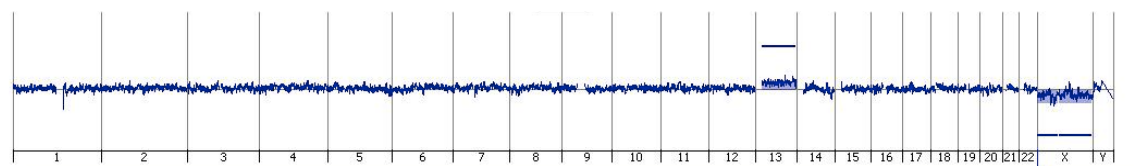

Cell C.3

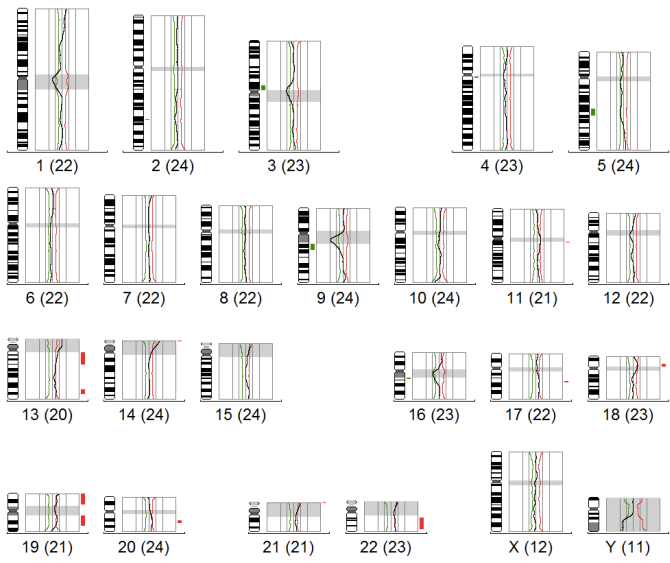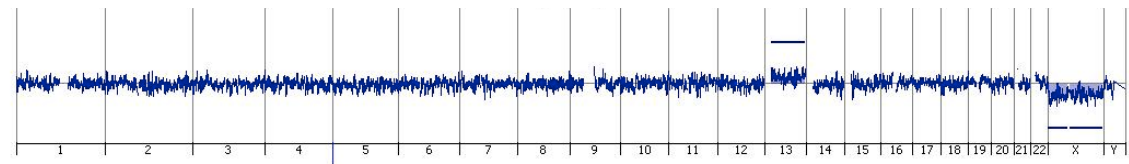

Supplement: Figure S1 — Oligonucleotide aCGH profiles obtained for the 15 fibroblasts analyzed. (PDF) [file pone.0113223.s001.pdf]

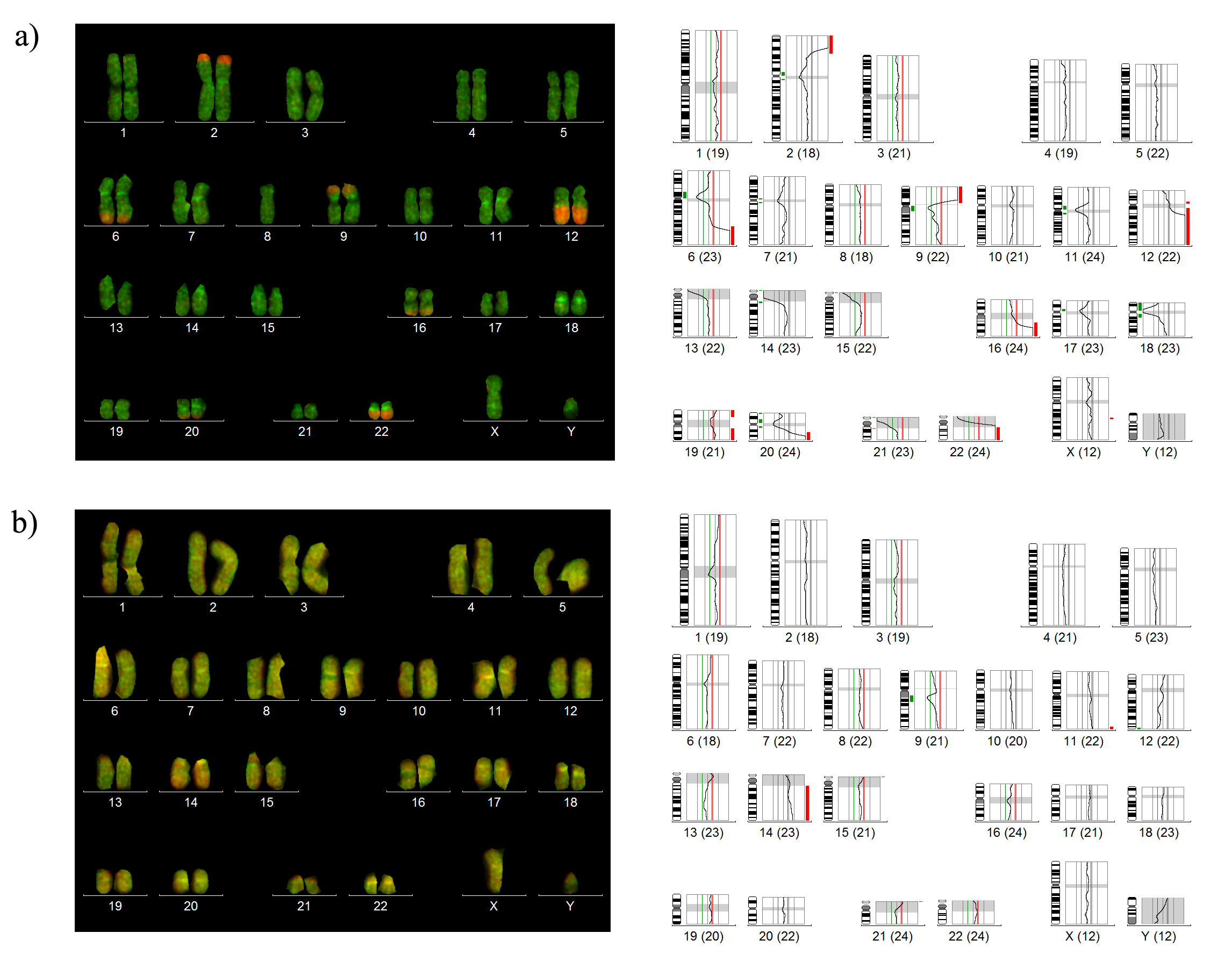

Supplement: Figure S2 — mCGH profiles obtained for E6 (Couple A) in a) one blastomere, and b) the whole, discarded embryo. (TIF) [file pone.0113223.s002.tif]
