# Supplementary material for: Oligonucleotide Arrays vs. Metaphase-Comparative Genomic Hybridisation and BAC Arrays for Single-Cell Analysis: First Applications to Preimplantation Genetic Diagnosis for Robertsonian Translocation Carriers
Source: PLoS One. 2014 Nov 21;9(11):e113223. doi: 10.1371/journal.pone.0113223 (PMC4240610; doi:10.1371/journal.pone.0113223)
Supplement: Table S1 — Cytogenetic results obtained in the PGD for couples A and B, with both males carrying a 45,XY,der(13;14)(q10;q10) Robertsonian translocation. (DOC) [file pone.0113223.s003.doc]

**Table S1.** Cytogenetic results obtained in the PGD for couples A and B, with both males carrying a 45,XY,der(13;14)(q10;q10) Robertsonian translocation.

| **C.E** | **Segr.** | **Agilent aCGH 8x60K** | | | **Agilent aCGH 4x180K** | | | **mCGH** | | |
| --- | --- | --- | --- | --- | --- | --- | --- | --- | --- | --- |
|  |  | SC | A | S | SC | A | S | SC | A | S |
| A.1 | Alt 2:1 | 46,XY | - | -4q24qter | 46,XY | - | -4q24qter | 46,XY | - | -4q25qter |
| A.2 | Alt 2:1 | 45,XX | -16 | -12q | 44,XX | -12, -16 | - | 44,XX | -12, -16 | - |
| A.3 | Alt 2:1 | 46,XY | - | -1q, +3q22.1qter, +6q22.2qter | 46,XY | - | -1q, +3q22.1qter, +6q22.2qter | 46,XY | - | -1q, +3q22qter, +6q22qter |
| A.4 | Alt 2:1 | 45,XX | -6 | -5q22qter, -8q, -10q | 44,XX | -6, -10 | -5q22qter, -8q | 45,XX | -6 | -5q22qter, -8q, -10q |
| A.5 | Alt 2:1 | 45,XX | -4 | - | 45,XX | -4 | - | 45,XX | -4 | - |
| A.6 | Alt 2:1 | 48,XXY | +22 | +2p24.1pter, +6q24.1qter, +9p21.2pter, +12q14.3qter, +16q22.2qter, +20q13.13qter | 48,XXY | +22 | +2p24.1pter, +6q24.1qter, +9p21.2pter, +12q14.3qter, +16q22.2qter, +20q13.13qter | 48,XXY | +22 | +2p21pter, +6q22.3qter, +9p, +12q, +16q, +20q |
| A.7^1^ | Alt 2:1 | 46,XY | - | - | 46,XY | - | - | 46,XY | - | - |
| A.8 | Adj 2:1 | 43,XY | -4, -8, -13 | -12q14.3qter | 43,XY | -4, -8, -13 | -12q14.3qter | 44,XY | -4, -13 | -8q11q22.1, -12q14.3qter |
| A.9^1^ | Alt 2:1 | 46,XY | - | - | 46,XY | - | - | 46,XY | - | - |
| B.1 | Adj 2:1 | 45,XX | -13, -17 | - |  |  |  | 45,XX | -13, -17 | - |
| B.2 | Adj 2:1 | 47,XX | +14 | - |  |  |  | 47,XX | +14 | - |
| B.3 | Adj 2:1 | 45,XY | -14 | - |  |  |  | 45,XY | -14 | - |
| B.4 | Adj 2:1 | 55,XXXYY | +3, +4, +5, +7, +13, +16, +XXY | +1p, +2p22.1pter, +2q22.3qter, +6q16.1qter, +10p, +18p, +18q22.1qter, +20q |  |  |  | 55,XXXYY | +3, +4, +5, +7, +13, +16, +XXY | +1p, +2p22.1pter, +2q22.3qter, +6q16.1qter, +10p, +18p, +18q22.1qter, +20q |
| B.5 | Alt 2:1 | 46,XY | +1, -3 | - |  |  |  | 46,XY | +1, -3 | - |
| B.6 | Adj 2:1 | N.A |  |  |  |  |  | 48,XXY | +13, +X | - |
| B.7 | Adj 2:1 | 45,X | -13, -X/Y |  |  |  |  | 45,X | -13, -X/Y |  |
| B.8^1,2^ | Alt 2:1 | 46,XY | - | - |  |  |  | 46,XY | - | - |

C.E: Couple.Embryo, Segr.: Meiotic segregation, SC: Sexual chromosomes, A: Aneuploidies, S: Segmental imbalances, N.A: not analysable. **^1^**Transferred embryos. **^2^**Pregnancy achieved.
