# Supplementary material for: Oligonucleotide Arrays vs. Metaphase-Comparative Genomic Hybridisation and BAC Arrays for Single-Cell Analysis: First Applications to Preimplantation Genetic Diagnosis for Robertsonian Translocation Carriers
Source: PLoS One. 2014 Nov 21;9(11):e113223. doi: 10.1371/journal.pone.0113223 (PMC4240610; doi:10.1371/journal.pone.0113223)
Supplement: Table S2 — Cytogenetic results obtained by mCGH from the discarded embryos of the PGD for couples A and B, with both males carrying a 45,XY,der(13;14)(q10;q10) Robertsonian translocation. (DOC) [file pone.0113223.s004.doc]

**Table S2.** Cytogenetic results obtained by mCGH from the discarded embryos of the PGD for couples A and B, with both males carrying a 45,XY,der(13;14)(q10;q10) Robertsonian translocation.

| **Couple.Embryo.Blastomere** | **Segregation** | **Cytogenetic results** |
| --- | --- | --- |
| A.1 | Alt 2:1 | 46,XY |
| A.2 | Alt 2:1 | 45,XX,-16 |
| A.3 | Alt 2:1 | 46,XY |
| A.4 | Alt 2:1 | 46,XX,+15q23qter |
| A.5 | Alt 2:1 | 46,XX |
| A.6 | Adj 2:1 | 47,XY,+14 |
| A.8 | Adj 2:1 | 46,XY,-13 |
| B.1.1 | Adj 2:1 | 44,XX,-13, -17, +1q, +16q |
| B.2 | Adj 2:1 | 47,XX,+14 |
| B.4.1 | Alt 2:1 | 46,XX,+2p,+5p15pter |
| B.6 | Adj 2:1 | 45,XY,-13 |
| B.7 | Adj 2:1 | 45,XY,-13 |
